# Review of cellular and molecular pathways linking thrombosis and innate immune system during sepsis

**Published:** 2010

**Authors:** Filip A. Konecny

**Affiliations:** a University of Veterinary Medicine and Pharmaceutical Sciences, Brno, Czech Republic; and St. Joseph Hospital and McMaster University, Ontario, Canada. E-mail: filkon@canada.com

**Keywords:** Inherited Immune System, Thrombin, PAR, TLR, Cell Cycle

## Abstract

Cellular and molecular pathways link thrombosis and innate immune system during sepsis. Extrinsic pathway activation of protease thrombin through FVIIa and tissue factor (TF) in sepsis help activate its endothelial cell (EC) membrane Protease Activated Receptor 1 (PAR-1). Thrombin adjusts the EC cycle through activation of G proteins (G12/13), and later through Rho GEFs (guanine nucleotide exchange factors), and provides a path for Rho GTPases mediated cytoskeletal responses involved in shape change and permeability of the EC membrane leading to an increase of leakage of plasma proteins.

At the same time, thrombin stimulates spontaneous mitogenesis by inducing activation of the cell cycle from G0-G1 to S by down-regulation of p27Kip1, a negative regulator of the cell cycle, in association with the up-regulation of S-phase kinase associated protein 2 (Skp2). After transport in cytoplasm, p27 Kip1 binds to RhoA thus prevent activation of RhoA by GEFs, thus inhibit GDP-GTP exchange mediated by GEFs. In cytoplasm, releasing factor (RF) p27-RF-Rho is able to free RhoA. P27 RF-Rho binds p27kip1 and prevents p27kip1 from binding to RhoA. Exposed RhoA is later able to increase the expression of the F-box protein Skp2, after its Akt triggered 14-3-3-β-dependent cytoplasm relocation. Skp2 increases cytoplasm ubiquitination-dependent degradation of p27Kip1. Additionally, after septic induction of canonical NF-kB pathway in EC through TLR4/IRAK4/TRAF/IkB, an IKKα dimer phosphorylates the p52 precursor NF-kB2/p100, leading to p100 processing and translocation of RelB/p52 to the nucleus. By controlling the NF-kB-RelB complex, IKKα signaling regulates the transcription of the Skp2 and correspondingly p27Kip1.

## The Role of Tissue Factor in Activation of Coagulation-Innate Immune System in Sepsis

**Figure 1:**
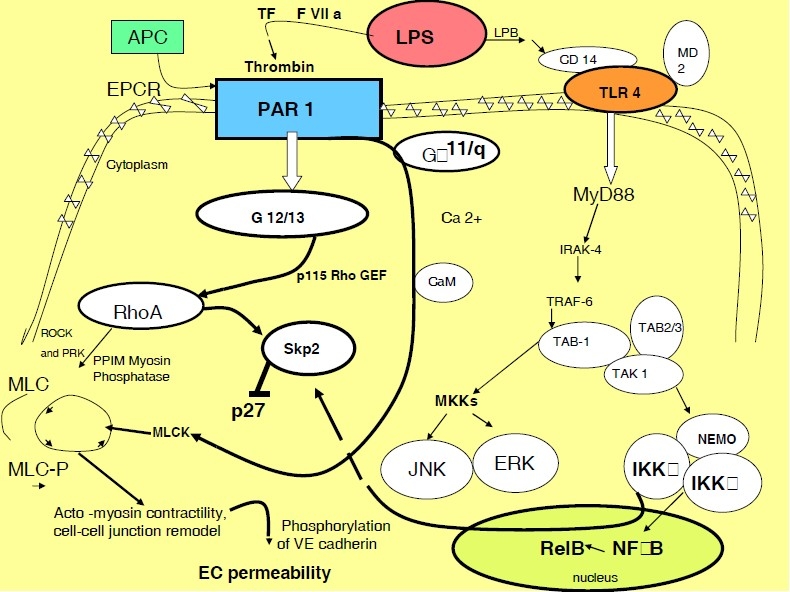
During innate immune activation via LPS-LPB-TLR4,^1^ a number of factors of the coagulation pathway are activated, creating the cooperative immuno-coagulative response. Extrinsic pathway activation of protease thrombin through FVIIa and tissue factor (TF) in sepsis help activate its EC membrane PAR-1 receptor.^2^ Reorganization of the endothelial cell (EC) cytoskeleton and cell adhesive complexes provides a structural basis for an increased vascular permeability implicated in the pathogenesis of sepsis. Thrombin through G-proteins (G 12/13 and G alpha 11/q)^2^ induces a rapid increase of the myosin regulatory light chain (MLC) kinase which results in actin-myosin interaction, stress fiber formation. Actin-myosin interaction leads to an increased EC permeability.^3^ MLCK inhibitors significantly attenuate the effects of thrombin on EC permeability and actin stress-fiber formation. As thrombin disrupts intercellular junctions, it creates formation of stress fibers and paracellular gaps by inhibition of Rho GTP-ase.^3^ Activated protein C (APC) is augmented with presence of EPCR that is present on ECs. Activation of PC by thrombin and thrombomodulin is restrained in sepsis. PAR-1 can be switched by APC, in presence of its receptor EPCR, or by thrombin from permeability protective to permeability enhancing.^4^ LPS: Lipopolysaccharide; GEFs: Guanine Nucleotide Exchange Factors; RhoA: Ras Homologue Family Member A; MLCK: Myosine Light Chain Kinase; CaM: Calmodulin; PLC: Phospholipase C; Skp 2: Sphase Kinase-Associated Protein 2; ROCK: Rho-Associated, Coiled-Coil Containing Protein Kinase; PRK: PKC Related Kinase; EPCR: Endothelial Protein C Receptor; TAB 1-TGF-beta: Activated Kinase 1/MAP3K7 Binding Protein 1; JNK: Jun N-Terminal Kinase; ERK: Extracellular Signal-Regulated Kinase.

In an organism, there are molecular and cellular components of coagulation and innate immune system sensing DAMPs (Danger Associated Molecular Patterns). Endogenous DAMPs (alarmins) and exogenous pathogen associated molecular patterns (PAMPs) are recognized by PRRs (Pathogen Recognition Receptors). PRRs can be divided into NLRs (NOD-like receptors), TLR (Toll-like receptors), and retinoic acid inducible gene I-like receptors or (RIG-I)-like helicases (RLHs). These receptors initiate the innate immune system and are later engaged in adaptive immune response. One of the molecules responsible for sepsis is endotoxin, constituent of the outer cell wall of gram-negative bacteria. Endotoxin consists of lipopolysaccharide (LPS). As LPS requires transfer to immune cells, the system of Tolllike receptor 4 (TLR4), CD14, and MD-2 represent the LPS receptor complex involved in the cellular recognition and signaling. Since it has amphiphilic structure, its spontaneous diffusion to cellular binding sites appears to be very slow for its tendency to form aggregates in aqueous solution. Lipopolysaccharide binding protein (LBP) significantly increases the transfer of LPS from aggregates to the CD14-TLR4 receptor complex (Figure 1).^1^ LBP is an acute-phase protein that recognizes and binds the lipid-A portion of LPS.

The rapid recognition of the antigen in a mammal’s circulation leads to the activation of the immune system. The innate immune system acts instantly followed by an early induced response, which does not lead to the lasting protective immunity.

During innate immune activation via LPS-LPB-TLR4, factors of the coagulation pathway are activated, creating the cooperative immuno-coagulative response. Activation of thrombin signaling through its PAR receptors constitutes coagulation pro-immuno activation. As the cascade progresses, cooperation of membrane bound factors i.e. TF (Tissue Factor) or factor Va, attachment of circulating cells to the damaged region ensures the cooperation of immune response with coagulation during sepsis. The short-lived thrombin in circulation and its only local production ensures the distinct area of such cooperative response. To fully understand this vast amount of variations of the activation of circulating cells with coagulation proteases, a multitude of settings has to be described to better understand the possible variations.

One such scenario in circulation is observed throughout activation of inactive innate surveyors DCs (Dendritic Cells) and inactive monocytes. Those cells are known to express membrane-bound TF, the transmembrane glycoprotein, and the cellular receptor for the zymogen factor VII and VIIa. When factor VIIa is bound to TF it activates cell signaling. Its binding potentiates intracellular calcium fluxes^2^ and activates mitogen-activated protein kinases (MAPK).^3^ It was assumed that TF is expressed only in tissues, and not at circulating blood cells and on vascular endothelium. Recently, Nijziel et al observed that in the quiescent state, monocytes express low levels of TF. After LPS stimulation, submicron TF-membrane positive particles, the fragments derived from activated and/or apoptotic cells become localized on its membrane.^4^,^5^ Thus the circulating phagocytic DCs and monocytic surveyors might represent a dual element of activation of coagulation and innate immune system during inflammation and sepsis. DCs are professional antigen-presenting cells, similar to other antigen-presenting cells i.e. macrophages and B cells. They function by entrapping and presenting the antigen to the T cells. DCs were for a very long time undistinguishable from the monocytes/macrophages due to the scarcity of its cell markers. After discovery of its surface markers, two major populations were detected. A set of population, which did not migrate to the periphery, and produce the interferon (IFN) was called plasmacytoid DCs. Migratory group, was called the non-lymphoid tissue migratory or lymphoid tissue resident DCs.

Monocytes in developing thrombus release TF, reaching its peak in about 100 s,^6^ in addition to the TF that already circulating in blood (100-150 pg per ml).^7^,^8^ Likewise, leukocytes in the circulation constitutively express TF.^9^ Additionally, the activated leukocytes release numerous mediators, such as cathepsin G and elastase, activating both the coagulation cascade and platelets.

After TF binds FVII/VIIa the complex acquires both procoagulant and signaling activities. As coagulation progresses through the TF:FVIIa complex, factor VII/VIIa initiates f. IX and X cofactor activation. Later, f. Xa converts prothrombin to thrombin, while thrombin induces transformation of fibrinogen into fibrin with help of f. XIa. Factor XIIIa helps with final fibrin cross-linking. Varieties of fibrinogen/fibrin products, as well as later products of fibrin degradation, are released into circulation, where they contribute to activation of TF. This circulating TF and its amount are now in the midst of an intense debate. Hoffman et al reported occurrence of TF throughout clots, where it was found to be incorporated into the clot margins.^10^ This suggests that circulating TF is integrated into clots. Butenas et al reported that extremely low levels of circulating TF are not likely to contribute to coagulation.^11^ Circulating low levels of TF and its role in activation of coagulation could be seen as one of the main building blocks interconnecting coagulation with the innate immune inflammatory activation.

Recently, the role of tissue migratory DC’s signaling in severe sepsis was discussed by Ruf.^12^ The non-lymphoid tissue migratory DCs belong to the most important circulating cells in LPS-sepsis, representing the intersection of coagulation and inflammation. In comparison, the unstimulated circulating plasmacytoid DCs expressing undetectable levels of TLR 4, while cardiomyocyte that express far more TLR 4 responds only weakly or not at all to LPS.^13^ In addition, Li et al in 2008 confirmed that ligands such as LPS, TNFα or CD40L are necessary during the maturation of the immature DCs.^14^ After LPS induced maturation, DCs expressed Protease Activated Receptors (PARs) 1 and 3, but not PAR 4.^14^ Thrombin is a vital protease responsible for the activation of DCs through DC’s PAR1-S1P3 (sphingosine 1 phosphate receptor 3) pathway. This coupling of coagulation and innate inflammatory response takes place in the draining lymphonodes, where DCs migrate after thrombin-PAR 1 activation.^12^,^15^ In addition, PAR 1 is cleaved by other serine proteases like activated protein C (aPC). At the same time that thrombin up regulates the proapoptotic genes; the aPC is counteracting thrombin by suppressing the NFκB downstream signaling.^16^

PARs belong to the G-transmembrane coupled protein receptors participating in two-way coagulation-inflammatory response. PARs are present on the cell membrane of numerous cells i.e. endothelial cells (ECs), platelets, leukocytes, neurons, and myocytes. They are known to be activated by the coagulation proteases such as thrombin, TF-FVIIa and TF-FVIIa-FXa complexes, and factor FXa.^17^ Thrombin exerts it cell activity partially through the G-protein coupled PAR receptors (PAR 1, 3, and 4); TF: VIIa and factor Xa and Trypsin, but not thrombin activate PAR 2.^18^ PARs help to activate the key signaling pathways of hemostasis, enabling thrombin to activate platelets, particularly PAR 4, which is in rodents essential for platelet activation by thrombin.

Interestingly, in PAR single-knockout (KO) mice (PAR 1, PAR 2, and PAR 4 KO) and in double-KOs e.g. PAR 1: PAR 2 and PAR 2: PAR 4 neither single PAR deficiency nor combined PAR deficiency (such as PAR 1: PAR 2 or PAR 2: PAR 4) had any significant role in mouse survival during LPS-induced endotoxemia.^19^ PAR (PAR 1-PAR 4) receptors expressed on circulating cells rather than on tissue cells i.e. cardiomyocyte, pneumocytes, neurons etc, at different stages of its activation and in different amounts, regulate the endotoxemic-coagualtion-innate inflammatory response through activity of its TLR’s. Thus the amount of TLR’s might not play a role in the final activation of the innate response, and rather the PARs cooperation with other circulating cells or vascular endothelium might activate the adaptive immune system. This leads to an overproduction of cytokines, which causes a loss of cardiomyocytes or pneumocytes. To further comprehend the condition(s) of both TLRs and PAR presence and its signaling capacities, a double KO mouse has to be generated. Moreover, the specific tissue KO mouse e.g. pneumocyte TLR 4 KO or the cardiac TLR 4 KO tissue specific, PAR 1 systemic KO mouse would be vital to study the coagulation-innate immune response in endotoxemia.

## The Role of Non-Phagocytic Platelets in the Initiation of the Thrombo-Immuno Response after the Induction of Sepsis

LPS exposure causes activation of coagulation and fibrinolytic systems, platelet consumption, reduced numbers of circulating lymphocytes and monocytes, and increased numbers of granulocytes. In 2006 Camerer et al, contrary to widespread belief, described in mice lacking PAR 4 (PAR 4 KO) that thrombin is not the key enzyme responsible for thrombocytopenia during endotoxemia. Moreover, its signaling in platelets is not necessary for endotoxin induced thrombocytopenia, and thrombin-induced platelet procoagulant activity is not important for thrombin generation.^19^

LPS proteins, Gram-negative and Gram-positive, including triacyl-cysteine-modified, can be included into common strong endotoxins. Biochemically, endotoxin proteins have an N-terminal cysteine residue that is modified by the addition of an ether-linked diacylglyceride, usually containing an N-terminal fatty acyl amide. Such modified proteins are powerful inflammatory agonists. When endotoxins are inoculated, rapid thrombocytopenia is observed. Unfortunately, there is limited publication characterizing LPS-induced platelet pathways and its relation to the innate immune system. The action of thrombin, based on the Camerer’ et al results, is not a key to the thrombocytopenia development.^2^,^3^

In 2003, Montrucchio et al found that LPS alone did not activate platelet aggregation. Rather the priming of platelets with the epinephrine, adenosine diphosphate or the arachidonic acid helped the aggregation.^20^ Results were confirmed in 2009 by Zhang et al by adding collagen or thrombin as the LPS-platelets aggregation enhancers.^21^

Platelet responses to thrombin include shape change, synthesis, release of the thromboxane A2,^22^ activation of the integrins,^23^ and binding of fibrinogen and von Willebrand Factor (vWf) to mediate the aggregation.^24^ Thrombin enhances the aggregation by different modes compared to collagen and is considered as one of the most potent platelet agonist. Thrombin binds to the platelet G-protein membrane receptor, causing platelet shape change an increase of cytosolic free Ca+, its degranulation, and aggregation. Varying concentrations of alpha-thrombin or by beta-and gamma-thrombin activates platelets through three thrombin receptors, PAR-1, PAR-4 and GPIb alpha. Other enzymatic reaction(s) takes place, rather than thrombin acting alone, in commencement of thrombocytopenia after LPS stimulation. However these confounding enzymatic, platelet priming reactions are unknown at this time; they could partially help to understand the Camerer’s data.

LPS is recognized by platelet immune Toll-like receptors. TLR 1, TLR 2, and TLR 6^25^ and TLR 4^26^,^27^ were identified as one of the cell associated receptors that play role in an early detection of the antigen circulating in blood. Human platelets activated by thrombin in the presence of Ca 2+ did not significantly affect the expression of TLR2 or TLR4 but significantly enhanced the expression of TLR 9.^28^ Thus the up regulation of TLR 9 in mouse platelets, challenged with LPS, suggests that other unknown TLRs might be derived from intracellular compartments.^28^

Platelets express the LPS-receptor-signaling complex, TLR 4, CD 14, MD2 and MyD88.^21^ In low concentration of platelet agonists (collagen or thrombin), in-vitro LPS enhances platelets aggregation. Further, in-vitro LPS induces ATP release, which is indicative of its dense granules secretion, but it is much less pronounced compared to thrombin ATP release, partially explaining LPS platelets signaling and aggregation insufficiency.

LPS stimulates platelet α (P-selectin, PF-4) and dense granules secretion. Amplification of aggregation occurs through the TLR4/MyD88-dependent mechanism and the cGMP/PKG dependent pathway.^21^ After the LPS stimulation thrombin is involved in the triggering of platelet’s P-selectin, the cell adhesive molecule (CAM), and its release from within α-storage granules to its surface. In addition, after stimulation of platelet’s TLR 2 by LPS, P-selectin surface expression and activation of platelet GP IIbIIIa increases. Integrin GP IIbIIIa is necessary for triggering the conversion of a dormant cell surface into the highly receptive one through its major agonists (collagen, fibrinogen, vWf), which are present in circulation via activation of the phosphoinositide 3-kinase (PI3-K)/Akt signaling pathway.^25^ The distal lectin-like domain of the P-selectin binds to a carbohydrate group presented on mucin-type glycoprotein i.e. P-selectin Glycoprotein Ligand 1 (PSGL-1) on the leukocytes. The post-LPS interaction through the TLR4/MyD88-dependent pathway^21^ is priming platelets to further interact with other members of the innate immune system. LPS primed monocytes and to lesser degree polymorpho-nuclear neutrophils (PMNs) are able to adhere on platelets.^20^ The role of P-selectin and its ligand (PSGL-1) in the activation of subgroups of leukocytes through platelet presentation and the up regulation on its membrane is rather controversial at this time. It suggests the role of P-selectin as a leukocyte-derived mediator, proposing that the activation of platelets by the LPS is mainly dependent on leukocytes, mostly monocytes as a result of CD14 and TLR4 engagement.^20^ Consequently after platelet activation, P-selectin is released and expressed on the cell surface. Binding of P-selectin to its ligand PSGL-1, expressed on all leukocytes, mediates the formation of platelet-leukocyte aggregates in the circulation and on damaged vascular surfaces on which platelets and fibrin have been deposited.^29^

Another mechanism of platelet thrombo-immune stimulation during the endotoxemia is through a secretion of Platelet-Activating Factor (PAF). PAF (1-O-alkyl-2-(R)-acetyl-sn-glyceryl-3-phosphonocholine) is a potent phospholipid mediator of many leukocyte functions. PAF induces platelet aggregation and vessel dilation. PAF is locally active and can be released from the adherent PMNs after the endotoxin or thrombin stimulation.^30^ Endotoxic stimulation induces production of a superoxide and the formation and shedding of the microparticles with bound PAF. The released material activates platelets, and they co-aggregates with endotoxin-stimulated PMNs.^30^ Recently the role of PAF in LPS induced endotoxemia was revisited by Jeong et al.^31^ They observed that dose dependent PAF injection significantly attenuated organ injury, after LPS injection, including profound hypotension, excessive polymorphonuclear neutrophil infiltration, lymphocyte apoptosis, severe Multiple Organ Dysfunctions (MODs), and death. PAF induced changes in the production of cytokines in response to LPS has two compensatory results. First it produces circulating pro-inflammatory cytokines (TNF-α, IL-1b, IL-12p70, and IFN) and, secondly it increases the production of compensatory anti-inflammatory cytokine IL-10.^31^

Platelet receptors and the understanding of its molecular functions and the downstream signaling pathways needs to be further explored. The use of pharmacological inhibition and knockout animals of known platelet receptors, adhesion molecules, and many signaling molecules might help to better comprehend the platelet role in thrombo-immune system. As an example, the case of TLRs and PARs on the platelet membrane, highlighting the role of platelets as both, the immunologic and thrombotic surveyors (thrombin activated platelets, in the presence of Ca 2+, significantly enhances the expression of TLR 9).^28^

## Thrombin Role in Cell Cycle and its Impact on Innate Immune System During Sepsis

LPS induction of TF causes generation of thrombin that is created after activation of TF: VIIa complex. TF: VIIa, factor Xa and trypsin activate G-protein coupled thrombin receptor PAR 2. It is the main protease of the coagulation cascade after its induction. Thrombin can induce the expression of pro-thrombotic factors i.e. TF and plasminogen activator inhibitor-1 (PAI-1).^32^ It enhances amplification of various chemokines (monocyte chemotactic protein-1)^33^ and cytokines (IL-6 and 8)^34^ and while inhibiting the expression of endothelial NO synthase (eNOS) and endothelin-1, it alters vascular tone.^35^ Thrombin exerts its cell activity partially through the G-protein coupled protease activated receptors (PAR 1, 2, and 4) limiting its activity to the place of its activation, while rapidly restricted by the action of protein C. Effects of Activated Protein C (APC) are mediated through the Endothelial Protein C Receptor (EPCR) and secondary through the activation of PAR1 by the APC-EPCR complex (Figure 1).^36^ On a constant basis APC loses the competition for PAR1 with thrombin.^37^ The cellular effects of APC in endotoxemia and severe sepsis are under continuing investigation, mostly because of its pleiotropism (i.e. coagulation, fibrinolysis, inflammation, immune cells, and vascular endothelial cells). New genetically engineered recombinant APCs (rAPC) are designed to control not only the anticoagulation, but also bleeding and apoptosis.

Normal expression of EPCR and PAR1 is an essential factor in mortality reduction by APC in mouse models of lethal endotoxemia. APC treatment of PAR1 KO mice challenged with LPS reproducibly prolonged the time of survival in a study by Kerschen et al.^38^ In addition, the selective direct thrombin inhibitor Hirudin decreases sepsis mortality and fibrin deposition in animal models of sepsis.^15^ Interestingly, partial KO mouse strains e.g. AT III+/-and (or) PC+/-challenged with endotoxin exhibit increased mortality and fibrin deposition.^39^,^40^

Generally, thrombin cellular responses consist of a regulation of the blood vessel diameter by an endothelial-dependent vasodilatation, triggering the calcium signaling and other responses in T lymphocytes.^41^ In endothelial cells (ECs), thrombin causes its shape change with an increase of a plasma permeability and edema.^42^ In a recent report by Hu et al in 2009, thrombin, after activation of its PAR 1 receptor, becomes a growth factor (GF) that can stimulate spontaneous mitogenesis by inducing activation of the cell cycle from G0-G1 to S by a down-regulation of p27Kip1. Its stimulating effect is equipotent with serum (a mixture of GF). Thrombin down-regulates p27Kip1, a negative regulator of the cell cycle, in association with the up-regulation of Skp2 and MiR-222, which decreases p27Kip1 by a different mechanism.^43^ Thus, in that setting, thrombin might operate posttranscriptionally by inactivation of p27Kip1. Thrombin can use protein complex SCF, Skp2-containing E3 ubiquitin ligase, and its cofactor cyclin dependent kinase-Cdk subunit1 (Cks1) (which recognize Thr187-phosphorylated p27Kip1) promoting its degradation by proteasome^44^ or other protein-protein interactions.^45^ Additionally the small protein Cks1 participates in p27Kip1 ubiquitination by increasing the binding affinity of Skp2 for p27Kip1.^45^ Thr187 phosphorylation directs p27Kip1 to the SCF Skp2 ubiquitin ligase complex (consisting of Skp2-Skp1-Cks1-Cul1-Roc1), which in turn promotes the polyubiquitination and degradation of p27Kip1.^46^ Through the activation of p27kip1 ubiquitination, thrombin actively promotes ECs mitogenesis.

**Figure 2:**
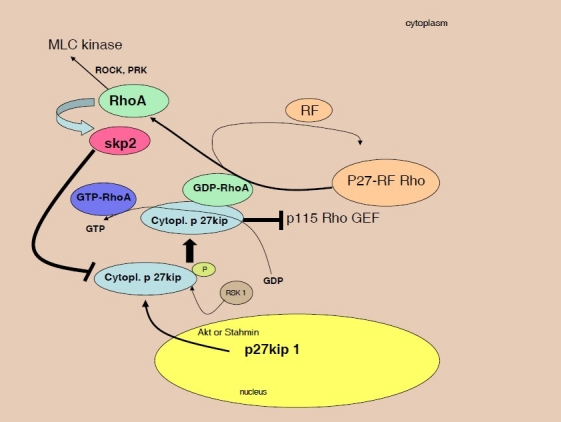
Disruption of cell barrier at the level of inter-endothelial junctions leads to an increase of leakage of plasma proteins and exudation of fluid in sepsis. Activation of the Rho GTP-ases was described in EC to prevent disassembly of inter-endothelial junctions and the increase in endothelial permeability. The Rho family GTP-ases (including Rho, Rac, and Cdc42) are crucial in regulation of permeability through spatio-temporal signaling.^49^ Rho proteins are activated through GDP-GTP exchange induced by guanine nucleotide exchange factors (GEFs) and are inactivated by GTPase-activating proteins. After transport in cytoplasm, p27 Kip1 binds to RhoA and prevent activation of RhoA by GEFs,^50^ thus inhibit GDP-GTP exchange mediated by GEFs.^50^ In cytoplasm, releasing factor (RF) of p27-RF-Rho was shown to be able to free RhoA. P27 RF-Rho binds p27kip1 and prevents p27kip1 from binding to RhoA. Exposed RhoA is later able to increase the expression of the F-box protein Skp2, after its Akt triggered 14-3-3-β-dependent cytoplasm relocation. Skp2 increases cytoplasmubiquitination-dependent degradation of p27Kip1. RSK1 (p90 ribosomal S6 kinase) phosphorylates p27, promoting p27-RhoA binding.^51^

Skp 2 belongs into a family of proteins containing the F box motif. The role of forkhead box M1 (FoxM1) in the molecular mechanisms of endothelial barrier repair following a vascular injury was recently studied by Zhao et al.^47^ In response to LPS, the endothelial cell restricted FoxM1 deficient mouse (FoxM1 CKO) displayed a significantly prolonged increase of the lung vascular permeability and the mortality due to severe pulmonary edema. Following the LPS-induced vascular injury, FoxM1 CKO lungs demonstrated an impaired cell proliferation in association with a sustained expression of p27Kip1 and decreased expression of cyclin B1 and Cdc25C. Zhao et al study suggests that impairment of proteins containing F box motif i.e. (FoxM1, Skp-2) might be an important determinant of persistent vascular barrier leakiness and edema formation associated with an inflammatory disease (Figure 2).

The activity of a PAR-1 receptor plays a significant role in thrombin signaling. PAR-1 is activated by a proteolytic activation and later discarded. Naive PAR-1 is delivered from the intercellular pool of PAR-1 to the endothelial cell (EC) surface in order to maintain thrombin responsiveness.^48^ At the beginning, thrombin locally activates its PAR-1 receptor on the EC and couples it to the members of small G proteins (G12/13, Gq, and Gi/z).^17^ Later, thrombin through Rho GEFs (guanine nucleotide exchange factors) provides a path for Rho GTPases mediated cytoskeletal responses that are involved in shape change and permeability of the EC membrane. The Rho GTPases are members of a Ras superfamily of GTP-binding proteins that act as molecular switches while cycling between inactive GDP-bound and active GTP-bound forms. This activity is controlled by e.g. guanine nucleotide exchange factors (GEFs) that catalyze exchange of GDP for GTP to activate the switch.^49^ Originally, Rho was identified as a signaling molecule that is involved in cellular functions. Rho was associated, for example, in cytoskeletal rearrangement and cytoskeleton organization during smooth muscle cell contraction, in the distinctive ability to induce specific filamentous actin structures in fibroblasts, and in platelet aggregation. Activation of Rho led to an assembly of contractile Actin-Myosin filaments, or actin rich filopodia or lamelliopodia.^49^,^50^ Recent studies support the original evidence and reveal RhoA and Rac1 activation by p27kip1.^51^ In addition, RhoA is able to increase the expression of the F-box protein Skp2 required for ubiquitination-dependent degradation of p27Kip1.^52^

P27Kip1 is a well-known cyclin-dependent kinase inhibitor (CDKI); a member of the Cip/Kip family. It is an essential cell cycle inhibitor that functions largely during the G0/G1 phase promoting the assembly of the cyclin D1-CDK4 complex and inhibits the kinase activity of the cyclin E-CDK2 complex in theG1-S phase. P27kip1 is contained in the nucleus, which functions largely during the G0/G1 phase, promoting assembly of the cyclin D1-CDK4 complex and inhibits the kinase activity of the cyclin E-CDK2 complex in the G1-S phase. P27Kip1 is also a phospho-protein with multiple Ser/Thr phosphorylation sites, including Ser-10, Ser-178, and Thr-187. In the latest review by Besson et al,^53^ authors showed that p27kip1 localization and its regulation depends on its amino acid phosphorylation. Thus e.g. the cytoplasmic localization of p27kip1 can be linked to Ser-10 phosphorylation that causes p27 Kip1 to be transported from the nucleus in G1 phase, leading to the progression of S/G2 phase.^51^,^54^ Ser-10 phosphorylation in dormant cells was proposed through Mirk/Dirk kinase, while in proliferating cells, there are reports implicating kinase interacting with stathmin (KIS), PKB/Akt, and extracellular signal-regulated kinase-2 (ERK2).^51^ Another phosphorylation site on p27 that regulates its localization in cytoplasm includes Thr-157 and Thr-198. The phosphorylation of Thr-187 directs p27Kip1 to a SCFSkp2 ubiquitin ligase complex and promotes its polyubiquitination and degradation. Phosporylation of Thr-157 or Thr-198 by PKB/Akt causes cytoplasmic localization of the phosphorylated p27 and its retention in cytoplasm.^55^ By promoting an association of p27 with 14-3-3 proteins, which prevents p27 from interacting with Importin α, p27 transport to the nucleus can be limited. These findings indicate that 14-3-3 protein suppresses importin α/β-dependent nuclear localization of Thr157-phosphorylated p27.^55^

The extent to which altered cellular localization modulates the many biological effects of Kip/Cip proteins is an area of considerable interest. In the case of p27 Kip phosphorylation by PKB/Akt or kinase-interacting stathmin (KIS), which mediates its translocation from the nucleus to the cytoplasm,^56^ cytoplasmic C-terminal end of p27kip1 binds RhoA^51^ and prevents activation of RhoA by GEFs.^53^ A novel protein was found by Hoshino et al,^56^ which might be able to augment activation of RhoA by releasing it from inhibition by p27kip1 and thus might regulate actin structures. This novel protein, p27RF-Rho (p27kip1 releasing factor from RhoA), binds p27kip1, thus preventing its binding to RhoA (Figure 2). Inhibition of p27 kip1 or its deficiency results in increased numbers of actin stress fiber formation, and focal adhesions. Disruption of the cell barrier during sepsis activated by thrombin at the level of inter-endothelial junctions leads to increase of leakage of plasma proteins and exudation of fluid in sepsis. The role of thrombin in modulation of levels of p 27 kip1 through e.g. RhoA that increases the expression of the F-box protein Skp2 required for ubiquitination-dependent degradation of p27Kip1 is in the midst of scientific debates.
